# Desmoplastic non-infantile ganglioglioma mimicking diffuse leptomeningeal glioneuronal tumor: precision diagnostics and therapeutic implications

**DOI:** 10.2340/1651-226X.2024.31720

**Published:** 2024-05-23

**Authors:** Pitt Niehusmann, Henning Leske, Vigdis Nygaard, Hege G. Russnes, Sen Zhao, Anna Latysheva, Ulrikke Straume Wiig, Birute Stankuniene, Aina Ulvmoen

**Affiliations:** a Department of Pathology, Oslo University Hospital, Oslo, Norway; b Division for Cancer Medicine, Oslo University Hospital, Oslo, Norway; c Department of Tumor Biology, Institute of Cancer Research Oslo University Hospital, Oslo, Norway; d Institute for Clinical Medicine, University of Oslo, Oslo, Norway; e Department of Cancer Genetics, Institute for Cancer Research, Oslo University Hospital, Oslo, Norway; f Department of Radiology, Oslo University Hospital, Oslo, Norway; g Department of Neurosurgery, Oslo University Hospital, Oslo, Norway; h Department of Paediatrics, Akershus University Hospital, Lørenskog, Oslo, Norway; i Department of Paediatrics, Oslo University Hospital, Oslo, Norway

**Keywords:** Brain tumor, HNRNPDL, BRAF fusion, DIG, neuropathology, precision medicine

## Introduction

Desmoplastic infantile ganglioglioma (DIG)/astrocytoma (DIA) are rare entities representing 0.4% of brain tumors and 1.25% of intracranial tumors in children [1]. These tumors typically present before the age of 24 months with clinical signs such as increased head circumference, bulging of the fontanelles, lethargy and sunset sign. There are few reports of non-infantile cases [2]. However, due to a lack of molecular data, the possibility of misclassification was considered and their true existence has been questioned [1]. In 2016, the diffuse leptomeningeal glioneuronal tumor (DLGNT) was introduced as separate entity into the WHO classification of central nervous system (CNS) tumors [3]. Radiologically, DLGNT typically shows a widespread diffuse leptomeningeal enhancement and thickening along the spinal cord, posterior fossa, brain stem and basal cisterns. In contrast, DIG/DIA presents typically as superficially located, large, contrast-enhancing solid and cystic tumor, where the solid component is frequently dural based. Here, we report a non-infantile patient suffering from a brain tumor with radiological features of a DLGNT, but convincing molecular pathological characteristics of a DIG/DIA.

## Case presentation

**Figure 1:**
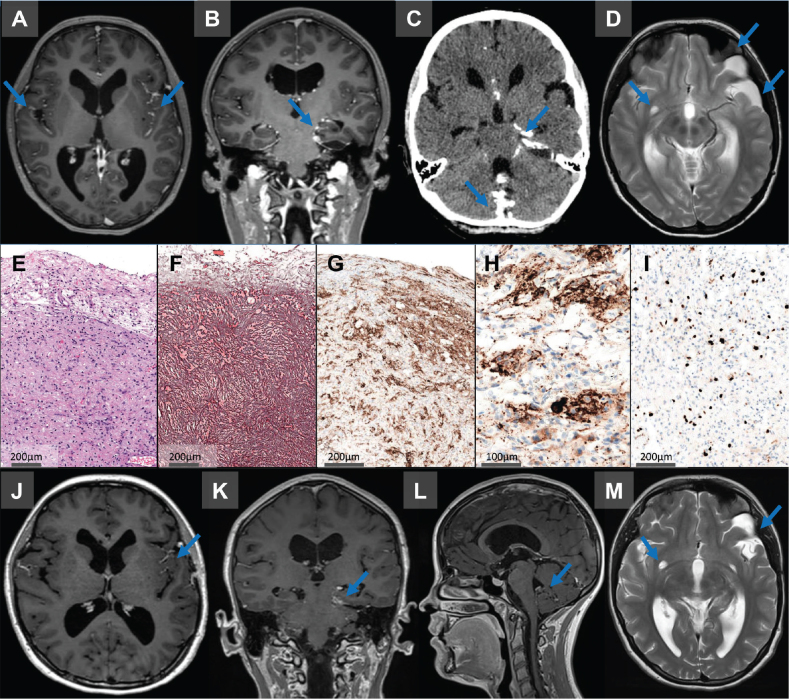
(A–D) MRI caput revealed a widespread leptomeningeal contrast enhancement, partially coinciding with calcifications, including spinal leptomeningeal tumor manifestations. In addition, a left temporomedial subpial cystic lesion with contrast enhancement in the cystic wall and a tetraventricular hydrocephalus was observed. (E–I) Neuropathological findings. Hematoxylin-Eosin staining (E) showed a moderately cellular tumor with a prominent desmoplastic leptomeningeal component, consisting of mainly fibroblast-like, spindle-shaped cells surrounded by reticulin-fibers (F). GFAP (G) highlighted scattered astrocytes within this component and synaptophysin showed a neuronal component with ganglionic differentiation (H). Ki67 immunohistochemistry demonstrated only moderate proliferative activity (I). (J-M) Six months after initiation of medical treatment MRI analysis revealed stable dimensions of the cystic lesion in the left temporal pole. However, there was a partial regression of enhancement observed within the cyst wall, as well as noticeable regression in pathological leptomeningeal enhancement.

A 17-year-old female patient presented to the children’s hospital with persisting headache, nausea and dizziness over the last 6 months. Clinical examination revealed a healthy-looking girl with age-appropriate features and intact neurological functions. The magnetic resonance image (MRI) revealed widespread leptomeningeal contrast enhancement, tetraventricular hydrocephalus and a left temporomedial subpial cystic lesion with radiological features suspicious for a DLGNT (see Figure 1A–D). Due to the disseminated character of the disease, complete resection was not possible and an open biopsy of the left temporal lesion was performed. Neuropathological work-up revealed a low-grade neuroepithelial tumor with a desmoplastic leptomeningeal component (see Figure 1E-I for histomorphological and immunohistochemical details).

Based on clinical, histomorphological and immunohistochemical features DLGNT and DIG were considered as main differential diagnoses, although the latter appeared less likely due to the disseminative character and the patient’s age. Molecular pathological panel analysis with the Oncomine Childhood Cancer panel (ThermoFisher) revealed no genetic alterations. Subsequently, the publicly funded Infrastructure for Precision Diagnostics – cancer (InPreD Norway) was commissioned for further comprehensive molecular work-up. TruSight Oncology 500 (Illumina) analysis revealed an *HNRNPDL*::*BRAF* fusion. The fusion product was assessed to be in-frame with an intact BRAF-kinase-domain (see Supplementary Figure 1). DNA methylation analysis (Infinium Methylation EPIC v.1.0, Illumina) revealed a matching score of >0.95 with the methylation class ‘desmoplastic infantile ganglioglioma/desmoplastic infantile astrocytoma’ (Heidelberg brain tumor classifier version 12.8). Chromosome arm 1p deletion, an essential diagnostic criterion for the diagnosis of DLGNT, was not detected. Based on these findings the biopsy was diagnosed as desmoplastic non-infantile ganglioglioma.

Due to the presence of a BRAF-fusion, treatment with the MEK inhibitor trametinib was initiated. Shortly after the onset of treatment, the initial symptoms disappeared, but the patient experienced skin toxicity, grade 2, in the form of acneiform dermatitis. The skin rash was controlled with additional antibiotic treatment using lymecyclin. Six months after initiation of trametinib administration an increased hair loss was observed. A noticeable regression of leptomeningeal enhancement was seen in the MRI at 3 months follow-up.

On last follow-up, 6 months after commencing trametinib, the patient has no new symptoms, but experience reduced quality of life due to the side effects of trametinib. MRI is without radiologically detected progression in neither supra- nor infratentorial regions (Figure 1J–M).

## Discussion

The integration of molecular findings represents a major leap in CNS tumor diagnostics. Based on tumor entity specific molecular alterations, cases with histomorphological and/ or immunohistochemical variation could be summarized into tumor groups that showed similar clinical behavior and outcome of the patients. Such molecular alterations rely on tumor specific mutations, chromosomal aberrations, gene fusions and methylome profiling. Therefore, the 2021 WHO classification of tumors of the CNS has partially introduced such molecular features as essential for the diagnosis of certain entities [1].

DIG and DLGNT are tumor entities with alterations that typically cause activation of the MAPK-signaling pathway. For DIG, the essential criteria in unresolved cases comprise either a ‘methylation profile of DIG/DIA or a BRAF or RAF1 mutation or fusion, occurring in the absence of homozygous deletion of CDKN2A and/or CDKN2B’ [1]. For DLGNT the molecular essential diagnostic criteria are chromosome arm 1p deletion and MAPK pathway alteration (mostly *KIAA1549*::*BRAF* fusion) or a methylation profile of DLGNT. *BRAF* fusion proteins, which signal in a dimerized and RAS-independent manner, have an intact *BRAF* kinase domain constitutively active due to replacement of the auto-inhibitory regulatory domain with a 5’ partner gene. Targeting downstream with MEK inhibitors represent a rationale to inhibit dimerized forms of *BRAF*-activation. Combined therapy of *BRAF* p.V600E-mutant pediatric low-grade glioma with type I BRAF inhibitor and MEK inhibitor has been approved. However, this combination is not recommended for the treatment of patients with tumors harboring BRAF fusions as type I RAF inhibitors are ineffective in this setting and may paradoxically enhance tumor growth [4].

Typically, the radiological pattern of both entities is rather distinct. The classic radiological features of patients with DIG comprise a superficially located solid and cystic as well as contrast-enhancing lesion. On MRI, the cystic component can be unilocular or multicystic and is T1 hypo- and T2 hyper-intense. The solid component is often dura based and hypointense on T1 and T2 with contrast enhancement.

In comparison, DLGNT shows typically widespread diffuse leptomeningeal enhancement and thickening along the spinal cord. Also, small cystic or nodular T2-hyperintense lesions at the subpial surface of the spinal cord and brain are common. There may be intraparenchymal lesions and there is often an obstructive hydrocephalus and associated periventricular T2-hyperintensity present.

Thus, the current case showed radiological features suggestive for a DLGNT. However, the neuropathological and molecular work-up revealed a desmoplastic component and an *HNRNPDL*::*BRAF* fusion not earlier described in DIG. BRAF-fusions have been detected in different primary brain tumors, particularly in pilocytic astrocytoma (WHO CNS grade 1). However, DNA-methylation analysis showed a clear match with DIG/DIA and there was no deletion of 1p detected. Some patients above the age of 2 years with brain tumors that showed histopathological characteristics of a DIG have been described previously, but these reports were from the pre-genetic era and lack molecular information [2, 5]. True existence of this entity has therefore been questioned [1]. Here, we describe for the first time a glioneuronal tumor in an adolescent patient with molecular and epigenetic findings that fulfill the essential diagnostic criteria for the classification as DIG.

Whereas no molecular alterations were detected using our standard next generation sequencing pipeline, i.e. Oncomine Childhood Cancer panel from ThermoFisher, the diagnostically relevant findings were obtained by using additional molecular analyses such as EPIC methylome analysis and the NGS panel TSO500, with the help of the publicly funded infrastructure for Precision Diagnostics – cancer (InPreD Norway).

This case underlines the need for advanced molecular analyses in the routine setting to allow WHO-conformed diagnoses and identification of possible targets for tailored therapy. It furthermore poses the need of reimbursement, for example as part of the public health care, to establish and maintain state of the art diagnostics.

## Data Availability

Data sharing appears not applicable to this article as it could compromise the individual privacy of the described patient. Data that is not subjected to this limitation are available from the corresponding author on reasonable request.
